# Energy-Efficient Protocol of Link Scheduling in Cognitive Radio Body Area Networks for Medical and Healthcare Applications †

**DOI:** 10.3390/s20051355

**Published:** 2020-03-01

**Authors:** Thien Thi Thanh Le, Sangman Moh

**Affiliations:** 1 School of Computing and Information Technology, Eastern International University, Binh Duong City 75114, Vietnam; thanhthien2003@gmail.com; 2 Department of Computer Engineering, Chosun University, Gwangju 61452, Korea

**Keywords:** wireless body area network, cognitive radio, electronic healthcare, link scheduling, energy efficiency

## Abstract

Wireless body area networks (WBANs) have become a new paradigm for electronic healthcare applications; for instance, they are used to efficiently monitor patients in real-time. In this paper, an energy-efficient link scheduling (ELS) protocol for cognitive radio body area networks (CRBANs) is proposed, which aims to minimize energy consumption in CRBANs, while achieving higher probabilities of successful transmissions with multiple CRBANs. The proposed ELS transmits packets in the common control channel to control transmission links amongst CRBANs to the gateway and vice versa. The transmissions of CRBANs to the gateway are scheduled at a specific time by the gateway in different data channels, according to the traffic priority of CRBANs. Packet delivery ratio, delay, and energy consumption are evaluated for multiple CRBANs via extensive simulation.

## 1. Introduction

**Figure 1:**
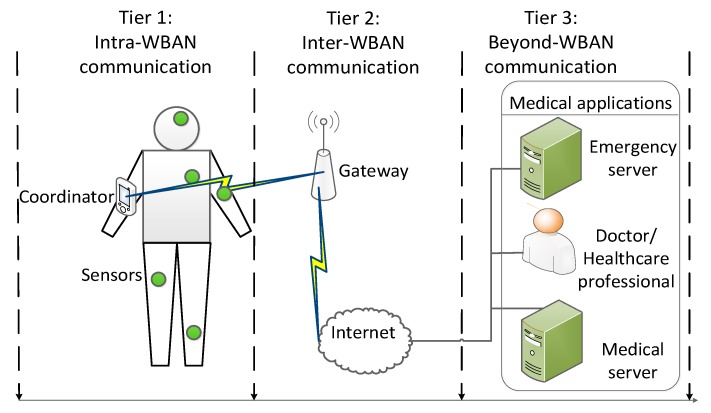
The typical cognitive radio body area network (CRBAN) architecture in medical application [2,3].

Wireless body area networks (WBANs) contain a coordinator and several biomedical sensors to collect vital signals in the human body using short-range wireless links. Recently, three physical layers for wireless communication in or around the human body have been recommended by the Institute of Electrical and Electronics Engineers (IEEE) standard for WBANs: Human body communication (HBC), narrow band, and ultra-wide band [1]. WBANs offer various applications in both medical and non-medical fields; for instance, monitoring emergency data and collecting the periodic data of patients in hospitals [2,3,4]. Figure 1 shows the typical architectural scenario for WBANs in medical applications in a three-tier communication model [2,3]. In the typical application at home, the elderly can wear some biosensors which will collect vital signals, such as blood pressure. Those vital signals are sent to the Internet gateway then forwarding to the doctor at the remote site. In another application at the hospital, some patients may send different types of vital data according to their sickness to the central gateway of the hospital. The central gateway will collect data from multiple WBANs and subsequently transmit the data to different servers, according to the type of traffic. For example, the emergency data packet from WBANs will be transmitted to the emergency server, while the monitoring data will be transmitted to the doctor. 

In a situation where multiple WBANs stay in the same place and transmit data to the gateway at the same time, interference may occur inevitably [5]. Therefore, the gateway needs to schedule the transmissions of WBANs with regard to the traffic priority before transmitting the data to different servers. In [3,4,5,6,7], cognitive radio (CR), which is essential to detect spectrum holes, can be implemented in healthcare and medical applications to reduce interference. As a gateway needs to gather information from multiple WBANs in the same area, the transmission of data packets in beyond-WBANs needs to be scheduled to ensure the quality of vital signals [8]. The characteristics of data transmission in WBANs are different from those in typical wireless sensor networks; thus, the CR platform for WBANs is introduced to achieve the required quality of service for healthcare applications in [9,10]. However, WBANs are densely deployed in a limited area, which leads to the need for a scheduling algorithm to achieve higher probabilities of successful transmissions. In [11,12], the scheduling algorithm aims to assign a predefined time or channel for the transmission of each CRBAN. In [11], the centralized scheduling algorithm is used at the gateway in order to assign data channels to CRBANs. In [12], the distributed scheduling algorithm is applied at CRBANs in which each CRBAN exchanges messages to its neighbors before scheduling the transmission of CRBANs. As a result, the transmission of each CRBAN is improved in terms of success ratio and latency. In another scenario, CRBANs may be in the same vicinity as other devices using the same channel [13]. Each CRBAN utilizes cognitive radio to change the working channel and avoid interference.

In addition, the security issue is a major factor in WBAN communication, which affects data privacy of individual patient [14]. For intra-WBAN communication, the data may be eavesdropped by the untrusted third-party or the attacker. The sender and receiver can define a feature set which will be used as an encryption key to protect sensitive data. For inter-WBAN communication, the access control mechanism is implemented to different users according to their legitimacy and roles, which aims to protect patient data. The interested reader may refer to [14] for more information because we do not pay much attention to secure communication link in the paper.

This paper is an extended version of our preliminary work in [11]. In this paper, we propose an energy-efficient link scheduling (ELS) protocol to schedule the transmissions of cognitive radio body area networks (CRBANs), which will be transmitted to different servers using different channels. Our algorithm aims to reduce the number of control packets in the network. The CRBANs could dynamically switch their working channel according to their monitored traffic priority level and the sensed idle channel. We assume that only the coordinator of CRBAN can sense idle channels and subsequently communicate with the gateway of scheduled channels. At first, the gateway schedules the transmission of CRBANs in the data channel with respect to the start of synchronization of each CRBAN; then, the coordinator of CRBANs will broadcast a beacon signal to its sensor nodes. The sensor nodes will change the data channel accordingly. It is assumed that the gateway has the capability of dual transmissions on multiple channels; i.e., the gateway receives data from CRBANs that are transmitted to different servers via different channels.

The main contributions of this paper are as follows:

- ELS allows each CRBAN to tune its working channel to the idle channel that is not occupied by the primary user (PU).
- ELS allows the gateway to schedule the transmission of multiple CRBANs into one data channel.
- ELS enables vital data from CRBANs to be aggregated at the gateway and forwarded to different medical servers.

The rest of this paper is organized as follows: In the following section, our system model of the target network and channels is introduced. In Section 3, the ELS protocol for CRBANs is presented in detail. In Section 4, the energy consumption per CRBAN is mathematically analyzed and evaluated in different network scenarios. The performance of the proposed ELS is evaluated via extensive simulation in terms of energy consumption, packet delivery ratio, and delay in Section 5. Finally, the paper is concluded in Section 6.

## 2. System Model

### 2.1. Network Model

**Figure 2:**
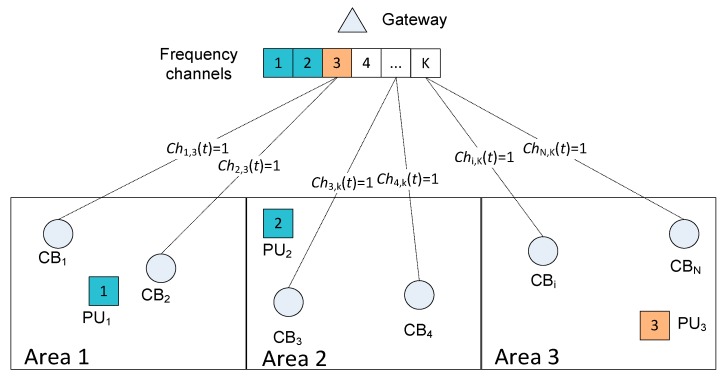
The network scenario.

The network is modeled for e-health applications, as shown in Figure 1. The network consists of one gateway, and *N* CRBANs denoted as *CB_i_*, 1 ≤ *i* ≤ *N*; each CRBAN consists of one coordinator, as well as *M* sensor nodes denoted as *s_ij_*, 1 ≤ *i* ≤ *N*, 1 ≤ *j* ≤ *M*. The data transmission time of each CRBAN is denoted as *T*(*CB_i_*), which is the total transmission time for *M* sensors. Assume that there are three different servers for three types of e-health applications, as shown in Figure 1, in which traffic priority is denoted as *p*3, *p*2, *p*1 for emergency server, doctor, and the medical server, respectively. The highest traffic priority is *p*3, and the lowest is *p*1. In *CB_i_*, the sensor node *s_ij_* generates packets *p_ij_* ∈ {*p*1, *p*2, *p*3}. The deployment of CRBANs and primary users is shown in Figure 2, in which several frequency channels are used in three separate areas. The primary users that are located in the same network occupy *K* non-overlapping orthogonal frequencies. The probability of primary user activity is modeled as a two-state Markov chain [13], where the states represent the idle and busy states of the channel. The probability that a primary user becomes idle is *p_idle_*, and the probability that a primary user becomes busy is *p_busy_*. We assume that the gateway senses the idle frequency by sensing the primary users’ activities. The notations used in this paper are summarized in Table 1.

We consider *N* CRBANs working on a spectrum of *K* channels containing *C_L_* licensed channels and *C_U_* unlicensed channels; i.e., *C_K_* = {*C_L_* ∪ *C_U_*}. Among *K* channels, the first channel of *C_U_* is chosen to be a common control channel, which is dedicated to the control packet transmission between the gateway and CRBANs. Each CRBAN will occupy one data channel. More than two CRBANs can occupy one data channel; the time offset is defined by the gateway. The operating time is divided in *T* superframes, and the operation of *CB_i_* in channel *C_k_* at superframe *t* is defined as *Ch_i,k_*(*t*) = 1. We assume that the channel sensing at the coordinator is implemented using an energy detection mechanism [9,15]. The received signal strength indicator (RSSI) is used to represent the energy level of received signals. The coordinator of each *CB_i_* senses the idle channels and creates a list of idle channels as follows:

*L_i_*(*t*) = {*C_k_* |*C_k_* ∈ (*C_L_* ∪ *C_U_*), RSSI(*C_k_*) < γ}. (1)

In the network scenario in Figure 2, the gateway maintains the working channel for the CRBANs, according to the primary users’ activities. We assume there are three areas; each area has several CRBANs and primary users. The gateway can sense the idle channels in the network. The gateway assigns idle channel *C_k_* to *CB_i_* denoted as *Ch_i,k_*(*t*) = 1 according to the primary users’ activities in each area. For example, *CB*_1_ can occupy channel *C*_3_ denoted as *Ch*_1,3_(*t*) = 1 in area 1 because primary user *PU*_1_ is working at channel *C*_1_.

### 2.2. Channel Model for CRBAN Transmission

The path loss model for intra-CRBAN transmission is defined in the IEEE 802.15.6 document as follows:

(2) $$PL(d)=a\cdot \log(d_{\left[mm\right]})+b+N,$$

where *d* is the distance between the transmitter and receiver in millimeters, *a* = 6.60 and *b* = 36.1 are parameters of the model, and *N* is the normally distributed variable with standard deviation σ*_N_* = 3.8 (in a hospital room scenario) [16]. 

The path loss model for beyond-CRBAN transmission follows the Friis model, which can be written as follows:

(3) $$PL(d)=PL_{0}+10n\log\left(\frac{d}{d_{0}}\right)+S,$$

where *d* is the distance between the coordinator of a CRBAN and the gateway, *PL*_0_ is the path loss at reference distance *d*_0_, and the path-loss exponent *n* ≥ 2. The shadowing *S* in dB is a random variable normally distributed with mean μ and standard deviation σ [8]. 

## 3. Energy-Efficient Link Scheduling Protocol for CRBANs

### 3.1. Energy-Efficient Link Scheduling

**Figure 3:**
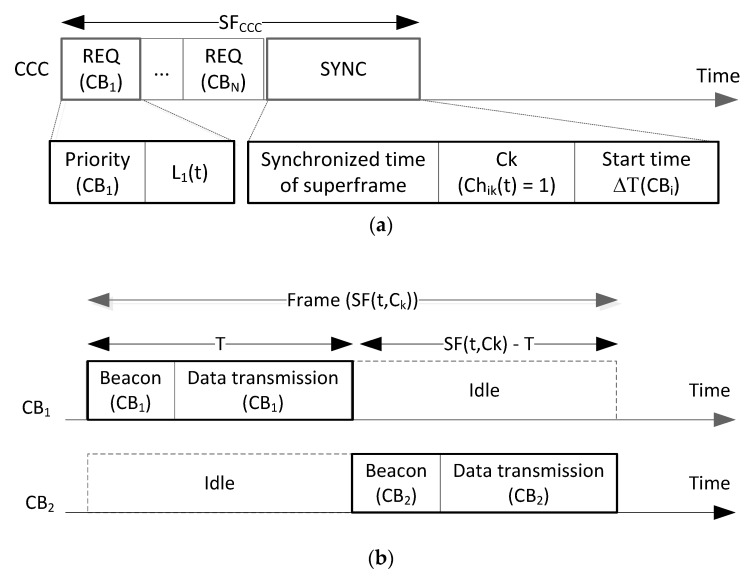
Superframe of multiple CRBANs: (**a**) Transmission in common control channel; (**b**) transmission in data channel *C_k_*.

The proposed ELS protocol is operated at the gateway. Assume that all CRBANs are synchronized, and the information obtained from the CRBANs is gathered at the gateway. The superframe of multiple CRBANs is shown in Figure 3. The superframe in common control channel, which is shown in Figure 3a, contains the request (REQ) and synchronization (SYNC). The REQ part is the length of REQ packets of *N* CRBANs; the SYNC part, which is sent by the gateway, contains the synchronized time or the start time of superframe, the indicator of channel *k* for each *CBi* called *Ch_ik_*(*t*), and the start time of CB*i*. In Figure 3b, the superframe of each CRBAN shows the frame in the data channel. Each CRBAN starts with the beacon signal, followed by the “data transmission” part and the “idle” part. The beacon is sent by the coordinator of the CRBAN to the sensor nodes. Next, the sensor nodes will transmit data to the coordinator in the duration of “data transmission”. Each CRBAN has the “idle” duration where it “remains silent” to avoid interference with the other CRBANs working at the same channel.

The gateway assigns the length of each CRBANs by the estimating priority value. Each CRBAN estimates the priority value, which is defined as follows:

(4) $$p(CB_{i})=\frac{\sum_{j=1}^{M}p_{ij}}{M},$$

where the priority value of each *s_ij_* is denoted as *p_ij_*, *p_ij_* ∈ {*p*1, *p*2, *p*3}. The list of detected idle channels is created at the coordinator of CRBAN (1). At the beginning of each frame, each CRBAN sends the *REQ* packet containing the priority value *CB_i_* denoted as *p*(*CB_i_*) and the list of detected idle channels *L_i_*(*t*) in the common control channel, as shown in Figure 3a. The priority value has two bits representing three different levels of traffic priority: {*p*1, *p*2, *p*3}. The gateway will schedule CRBANs at different channels according to the priority of traffic, as shown in Algorithm 1, and subsequently broadcast the schedule in the SYNC packet in the common control channel, as shown in Figure 3a. The SYNC packet contains the synchronized time of superframe, the data channel for CRBAN *Ch_i,k_*(*t*) = 1, and the Δ*T*(*CB_i_*) is the start of a superframe of *B_i_* after the synchronized time of superframe.

The gateway finds the CRBANs containing the same idle channels: *SL_k_*(*t*) = {*SL_k_*(*t*) ∪ *B_i_* | *C_k_* ∈ *L_i_*(*t*), 1 ≤ *i* ≤ *N* }. Assume that the CRBANs in *SL_k_*(*t*) will share the same channel *C_k_*, and the gateway calculates the length of frame for each CRBANs as follows:

(5) $$T(CB_{i})=\frac{SF(t,C_{k})}{L},$$

where *L* is the number of CRBANs in *SL_k_*(*t*), and *SF*(*t*,*C_k_*) is the maximum length of superframe in which *SF*(*t*,*C_k_*) ≤ *T_delay_*, *T_delay_* is the maximum latency of data [7].

In Figure 3b, each CRBAN occupies one data channel for intra-CRBAN transmission, as well as transmitting data to the gateway. In data channel *C_k_*, the length of a superframe contains multiple frames of CRBANs that occupy the same channel *C_k_*, as shown in Figure 3b. The frame of each CRBAN consists of a beacon signal and length of data transmission for intra-CRBAN transmission. The intra-CRBAN transmission is shown in Algorithm 2.

In Algorithm 1, after receiving the *REQ* packets from CRBANs, the gateway starts the energy-efficient scheduling to obtain the schedule for each CRBAN. The gateway will search for CRBANs containing the list of idle channels (as shown in line 1 in Algorithm (1). The gateway will search for each idle channel *C_k_* and assign the list of CRBANs to the sub-list *SL_k_*(*t*) which is empty, as shown in line 2; The *start_time* Δ*T* is equal to the start of the superframe or the synchronized time of superframe in line 3; The length of frame for each CRBAN, as shown in line 4. In line 5, the gateway finds the sub-list of CRBANs that sense the idle channel *C_k_*. The gateway chooses the CRBAN with the highest traffic priority, as shown in line 6 From line 7 to line 12, if the CRBAN with the highest priority value is not scheduled, the gateway updates the data channel of *CB_i_* to the current channel *C_k_*, as shown in line 8, and the start_time of *CB_i_*, as shown in line 9. As *CB_i_* is scheduled, the gateway removes *CB_i_* out of *SL_k_*(*t*), as shown in line 9, and updates the *start_time*, as shown in line 10. The SYNC packet is added with the information of *CB_i_*, as shown in line 11. From line 12 to line 14, if *CB_i_* is already scheduled, the gateway skips this step. The algorithm ends when all CRBANs are scheduled with the value *Ch_i,k_*(*t*) = 1 and *start_time* Δ*T*(*B_i_*)>0. The gateway broadcasts the SYNC in line 18.

**Algorithm 1.** Energy-efficient link scheduling at the gateway.**Input:***N* CRBANs, List of idle channels *C_k_*, and synchronized time of superframe T_0_ **Output:** List of data channel and the *start_time* for *N* CRBANs
1. **For** each *C_k_* ∈ *C_U_* ∪ *C_L_*
2.   Find the sublist of CRBANs: *SL_k_*(*t*) = {*SL_k_*(*t*) ∩ *B_i_* | *C_k_* ∈ *L_i_*(*t*), 1 ≤ *i* ≤ *N*} 
3.   Assign Δ*T* = *T*_0_
4.   Calculate *T*(*CB_i_*) as in (4)
5.    **For** each *CB_i_* ∈ *SL_k_*(*t*)
6.     Find *CB_i_* so that *CB_i_* has the highest priority value *p*(*CB_i_*)
7.     **If** (*CB_i_* is not scheduled)
8.        Set data channel for *CB_i_*: *Ch_ik_*(*t*) = 1
9.        Set *start_time* for *CB_i_*: Δ*T*(*CB_i_*) = Δ*T*
10.       Remove *CB_i_* out of *SL_k_*(*t*)
11.       Update Δ*T =* Δ*T*(*CB_i_*) + *T*(*CB_i_*)
12.       SYNC adds {*Ch_ik_*(*t*) = 1, Δ*T*(*CB_i_*)} 
13.     **Else**

14        Continue
15      **End If**
16.    **End For**

17. **End For**

18. Broadcast SYNC

### 3.2. Intra-CRBAN Data Transmission

We deploy the time division multiple access (TDMA) transmission for intra-CRBAN transmission, according to the IEEE 802.15.6 standard. The TDMA schedule of sensor nodes is executed (as shown in Algorithm 2) at the coordinator. The coordinator broadcasts the beacon packet, which includes the schedule of sensors, as well as the start time of transmission of each sensor. The coordinator finds the sensor node with the highest traffic priority, and subsequently adds it to the schedule (lines 3 to 4 in Algorithm 2). Next, the transmission time of the sensor is assigned, as shown in line 6. The transmission time of the next slot is defined, as shown in line 7. The coordinator already removes the sensor *s_ij_* from the set of unscheduled sensors, as shown in line 8. The algorithm ends when the transmissions of all sensors are scheduled into the TDMA frame. The sensor node *s_ij_* receives the beacon and starts the transmission at the *start_time*(*s_ij_*) in the beacon packet.

**Algorithm 2.** Intra-CRBAN data transmission.**Input:** Information of data channel and start_time of *CB_i_* {*Ch_ik_*(*t*) = 1, Δ*T*(*B_i_*)}, length of frame *T,* time slot for data transmission *t_s,_* set of unscheduled sensors *s_ij_* ∈ *CB_i_*
**Output:** Data transmission schedule of the sensor nodes
1. Assign *schedule* = ø 
2. **For** each *s_ij_* ∈ *CB_i_*
3.  Find the *s_ij_* so that (*p_ij_*)*_max_*
4.  Add *s_ij_* to the schedule: schedule = {*s_ij_*}
5.  Assign *start_time*(*s_ij_* ) = ΔT(B_i_)
6.  Update *current_time* = Δ*T*(*B_i_*) + *t_s_*
7.  Remove *s_ij_* out of *CB_i_*
8. **End For**

### 3.3. Link Scheduling Example in Multiple CRBANs

**Figure 4:**
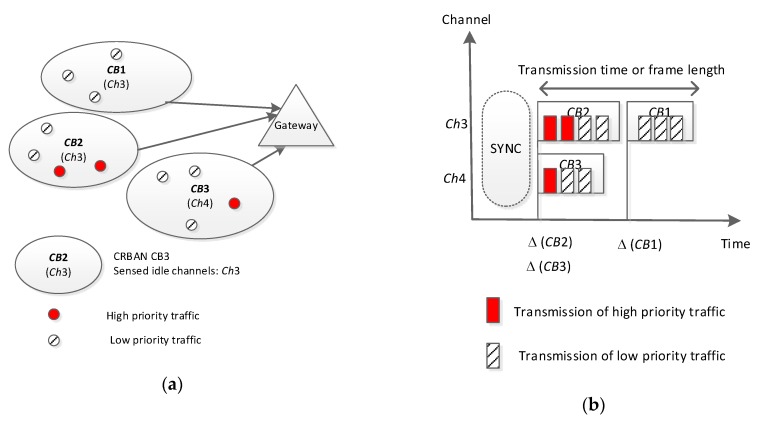
An example of scheduling multiple CRBANs: (**a**) network scenario and (**b**) schedule of data transmission.

An example of the ELS protocol is shown in Figure 4. As shown in Figure 4a, the network consists of one gateway and three CRBANs. Each CRBAN occupies a channel (referred to as “current working channel”) for data transmission. Assume that each CRBAN senses the idle channels with regard to the PU’s status. As a result, CRBANs create a list of idle channels according to the presence of PUs. Each CRBAN has several sensor nodes with different levels of traffic priority. The gateway schedules the transmission of three CRBANs, as shown in Figure 4b. CRBAN *CB*1 has the lowest priority, while *CB*2 has the highest because it contains two sensor nodes with high priority traffic. In Figure 4b, *CB*1 and *CB*2 occupy channel *Ch*3. As *CB*1 has a lower priority than *CB*2, the transmission of *CB*1 will be scheduled when *CB*2 finishes its transmission. In contrary, because *CB*3 does not interfere with other transmissions in *Ch*4, *CB*3 can occupy the transmission anytime.

### 3.4. ELS Evaluation in Different Network Scenarios

**Figure 5:**
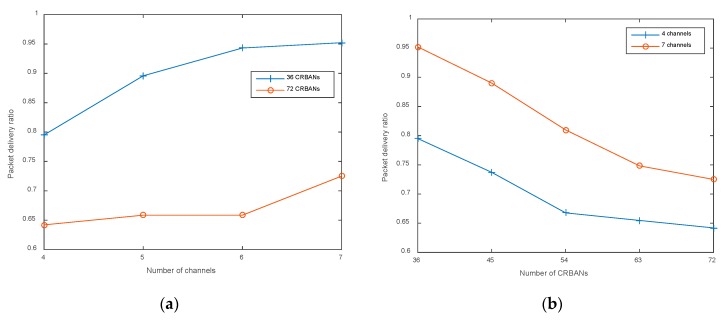
Packet delivery ratio: (**a**) varying the number of channels; (**b**) varying the number of CRBANs.

In this section, we examine ELS in the different network scenarios in terms of packet delivery ratio and latency; the results show that ELS performs well in the dense network with an acceptable success ratio and latency. For example, in the case of 72 CRBANs, multiple CRBANs can occupy a channel, which leads to a shorter superframe for intra-CRBAN transmissions; as a consequence, PDR decreases, as shown in Figure 5a. As shown in Figure 5b, PDR decreases with an increase in the number of CRBANs. However, if the number of channels in the network is large, the probability of idle channels in CRBAN transmissions is high. As a result, the PDR in the case of seven channels is higher than that of four, while varying the number of CRBANs. As a consequence, the PDR is lower than that of 36 CRBANs.

**Figure 6:**
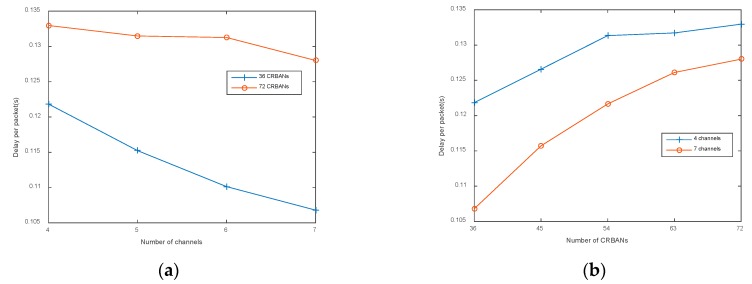
Delay per packet: (**a**) varying the number of channels; (**b**) varying the number of CRBANs.

As latency is an important parameter to evaluate the quality of service, we examine ELS in terms of delay per packet in different network scenarios as follows: Delay per packet is considered the latency until the packet arrives at the gateway, according to the generated time at the CRBAN. If the network density is unchanged, the delay per packet is decreased when using more channels to schedule the transmission of CRBANs, as shown in Figure 6a. The network density increases proportionally to the delay per packet, as shown in Figure 6b. In addition, the average delay per packet in the dense network is always higher than the one in the scattered network. However, the delay per packet is below 200 ms, which satisfies the requirement for medical applications.

## 4. Energy Consumption Analysis

### 4.1. Energy Consumption per CRBAN

To calculate the total time of packet transmission in a common control channel, we calculate the length of *REQ* and *SYNC* as follows. First, we assume that the length of priority value is 2 bits, which represents the third level of priority traffic. The length *L_i_*(*t*) is equal to the number of channels in the network. We assume that the total number of channels is 14, according to the number of channels in the IEEE 802.15.6 standard [1]. The total number of bits for *REQ* is 16 bits, which is equivalent to 2 bytes. The time length of REQ is denoted as *t_REQ_* = 2 bytes/(data rate). Total time length of REQ for *N* CRBANs is calculated as follows

*T_TEQ_* = *N* × *t_REQ_*, (6)

We assume that the energy consumption at the coordinator of a CRBAN is calculated in one successful superframe. The transmission of one CRBAN is considered successful if *CB_i_* is scheduled into the data channel after sending *REQ* packets to the gateway. 

The energy consumption for transmitting is calculated as follows:

(7) $$E_{tx}(total)=\frac{n_{Beacon}+n_{REQ}}{T_{Tx}}E_{Tx},$$

where *n_Beacon_* is the number of bits in beacon signals in the data channel, *n_REQ_* is the number of bits in REQ signals in common control channel, *T_Tx_* is the total transmitting time, and *E_Tx_* is the energy consumed during transmissions.

The energy consumed during receiving signals is calculated as follows:

(8) $$E_{rx}(total)=\frac{n_{SYNC}+T-Beacon}{T_{Rx}}E_{Rx},$$

where *n_SYNC_* is the number of bits in SYNC signals in common control channel, *T* is the number of bits of a superframe in the data channel, *n_Beacon_* is the number of bits in beacon signals in the data channel, *T_Rx_* is the total receiving time, and *E_Rx_* is the energy consumed during transmissions.

The energy consumed during channel sensing is calculated as follows:

(9) $$E_{sense}(total)=\frac{K}{T_{sense}}E_{ss},$$

where *E_ss_* is the energy consumed during sensing channels, *K* is the total number of channels, and *T_sense_* is the total time taken for sensing channels.

The energy consumed during channel switching is calculated as follows:

(10) $$E_{sw}(total)=\frac{Switch\_Ch_{CCC}+Switch\_Ch_{Data}}{T_{switch}}E_{Switch},$$

where *Switch_Ch_CCC_* is the length of switching to the common control channel for beyond-CRBAN communication with the gateway, *Switch_Ch_Data_* is the length of switching to data channel for intra-CRBAN communication, *E_Switch_* is the energy consumed during switching channels, and *T_switch_* is the total time taken to switch channels. 

The total energy consumption at the coordinator is defined as follows:

(11) $$E_{consump}\left(total\right)=E_{tx}(total)+E_{rx}(total)+E_{sw}(total)+E_{sense}(total),$$

where *E_tx_*(*total*), *E_rx_*(*total*), *E_sense_*(*total*), and *E_sw_*(*total*) are defined as in Equations (7)–(10), respectively.

### 4.2. Energy Consumption per CRBAN in Different Network Scenarios

**Figure 7:**
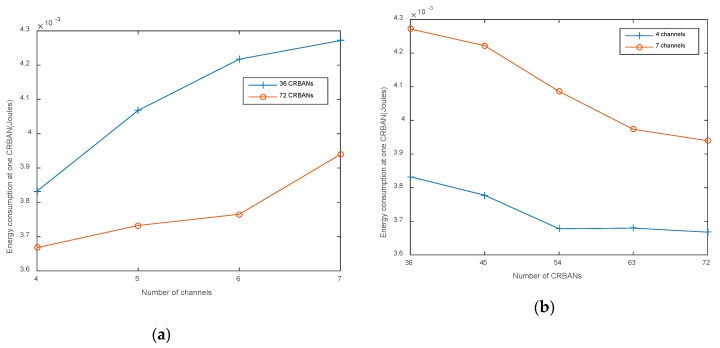
Energy consumption: (**a**) varying the number of channels; (**b**) varying the number of CRBANs.

The energy consumed at one CRBAN is calculated by Equation (11) in Section 4.1. CRBANs consume more energy when increasing the number of CRBANs and the number of channels, as shown in Figure 7a,b, respectively. In Figure 7a, when the network density is unchanged, the usage of more channels for transmission increases PDR, which leads to an increase in energy consumption. If the network density increases, more CRBANs will be scheduled into one channel, which leads to the degradation of PDR, as shown in Figure 7b. As a result, energy consumption per CRBAN decreases, due to a decrease in the number of successfully received packets.

## 5. Performance Evaluation

### 5.1. Simulation Environment

In this section, the proposed ELS protocol is evaluated using Matlab simulator and subsequently compared to existing scheduling schemes. We deploy the network under evaluation in a hospital for scheduling multiple CRBANs [3]. The network area is divided into nine rooms [3]; each room has one PU and several CRBANs. We compare ELS to spectrum-aware priority-based link scheduling (SPLS) algorithm [12], and channel selection algorithm for multiple WBANs and the applications of the Internet of Things (IoT) [13]. In [12], the SPLS algorithm aims to assign CRBANs to different channels. In [13], the channel selection algorithm will select the idle channel for WBAN transmission to avoid interference among IoT devices and WBANs. In this paper, “IoTWBAN” represents the channel selection algorithm for CRBANs [13].

At each CRBAN, the sensor nodes generate packets which are categorized into three different priority levels {*p*1, *p*2, *p*3}, where *p*3 denotes the highest traffic priority. According to [7], packet size is chosen in the range of 100 to 250 bytes, and the threshold of latency *T_max_* is set to 250 ms. The beyond-WBAN’s transmission rate is set to 500 Kbps, while the intra-WBAN’s is set to 250 Kbps [7]. The packet arrival rate is set to 2 p/s. The path loss parameters are set as follows: *d*0 = 1cm, *n* = 3.23, μ = 0, and σ = 4.85 [12]. Transmission bandwidth is 1 MHz and transmission noise *N*_0_ = −94 dBm. The best-case scenario is that each CRBAN could occupy one idle channel for data transmission; therefore, we assume that the minimum number of CRBANs is equal to the minimum number of idle channels, as shown in Table 2. We vary the number of CRBANs in the network area and the total working channels. In the first case, we vary the number of CRBANs in two scenarios of four and seven channels. In the second case, we vary the number of channels when the number of CRBANs is four and eight CRBANs per area.

### 5.2. Simulation Results and Discussion

#### 5.2.1. Energy Consumption at CRBANs

**Figure 8:**
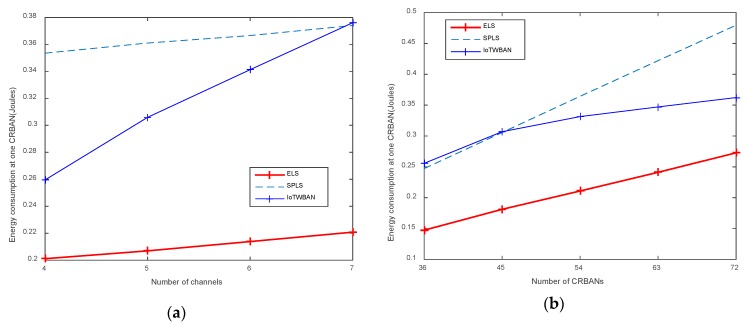
Energy consumption at one CRBAN: (**a**) varying the number of channels; (**b**) varying the number of CRBANs.

**Figure 9:**
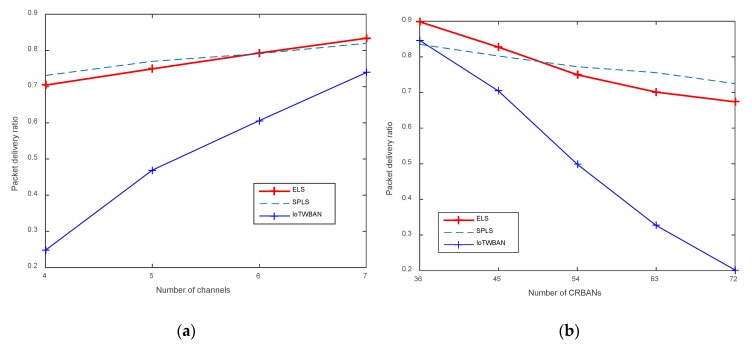
Packet delivery ratio of different scheduling algorithms: (**a**) varying the number of channels; (**b**) varying the number of CRBANs.

In Figure 8a,b, energy consumption at CRBANs increases proportionally to the number of channels and network density (i.e., the number of CRBANs). Our proposed ELS algorithm consumes less energy than the existing SPLS and IoTWBAN. As the ELS algorithm allows CRBANs to sense idle channels before switching to a new working channel, less energy is consumed during switching. However, as the PDR of ELS is high (as shown in Figure 9), the number of retransmissions is low, and less energy is consumed by ELS. The energy consumption of SPLS and IoTWBAN is similar when using a large number of channels or during a scattered deployment of CRBANs.

#### 5.2.2. Packet Delivery Ratio

Packet delivery ratio (PDR) is the ratio of the number of successfully received packets at the gateway to the number of the generated packets at CRBANs. As shown in Figure 9a, PDR increases proportionally to the number of channels. ELS and PSLS perform better than IoTWBAN; the reason is that, in ELS and SPLS, the channel selection algorithm is used to select one idle channel, as well as schedule the transmission of multiple CRBANs in one channel. On the other hand, PDR decreases in ELS, SPLS, and IoTWABN if we increase the number of CRBANs, as shown in Figure 9b. SPLS and ELS achieve high and similar PDRs; in a dense network of 72 CRBANs, PDR is about 70%, which is acceptable. However, IoTWBAN has the lowest PDR because CRBANs need to select an idle channel without interfering to other IoT devices. If the number of IoT devices is high, the CRBANs will content with IoT devices, which may cause a less number of successfully received packets.

#### 5.2.3. Delay per Packet 

**Figure 10:**
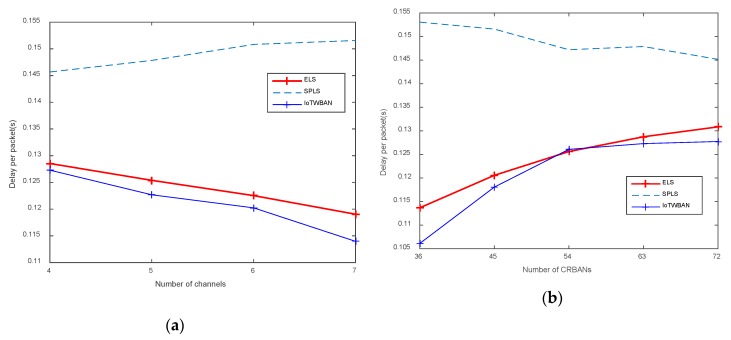
Delay per packet: (**a**) varying the number of channels; (**b**) varying the number of CRBANs.

As in Figure 10a, packet delay is inversely proportional to the number of channels. Our proposed ELS algorithm performs better than SPLS. However, ELS’ latency is slightly higher than IoTWBAN’s; the reason is that the ELS algorithm requires CRBAN to sense the new idle channel, which may be different from the current working channel. Therefore, CRBAN needs to switch to another channel to continue its transmission, possibly resulting in longer delays. As shown in Figure 9a, the latency is less than 130 ms, which is acceptable for most applications. Although the latencies are low in multiple available channels, they tend to increase proportionally to the number of CRBANs, as shown in Figure 10b. As multiple CRBANs occupy one channel, the latency will increase, and ELS performs slightly better than IoTWBAN and better than SPLS.

## 6. Conclusion

Energy consumption is vital to ensure the longevity of CRBANs in medical applications. It is necessary that WBANs consume low energy, while transmitting data efficiently in medical applications. In the proposed (ELS) algorithm, the licensed frequency bands are opportunistically utilized by the sensor nodes with cognitive radio. Each CRBAN is scheduled into a specific channel with a predefined offset time. As a result, energy consumption is significantly reduced, due to a decrease in the number of control packets, as well as the collisions between CRBANs. It can be reasonably inferred that the proposed scheme reduces energy consumption, while mitigating interference. When compared to other existing link scheduling algorithms, ours achieves the lowest value in terms of energy consumption, with neither decreasing packet delivery ratio nor increasing latency. In the future, we will improve this work to control link scheduling autonomously in accordance with different types of networks. We will also consider the impact of security attacks on system performance as our possible future work as well.

## Figures and Tables

**Table 1:** Table of notations.

Notation	Explanation
*CB_i_*	CRBAN index *i*
*s_ij_*	Sensor node index *j* of CB_i_
*p_ij_*	Traffic priority of *s_ij_*: *p_ij_* ∈ {*p*1, *p*2, *p*3}
*L_i_*(*t*)	List of idle channels of *CB_i_*
*C_k_*	Channel index *k*, *C_k_* ∈ (*C_L_* ∪ *C_U_*)
*C_L_*	Set of licensed channels
*C_U_*	Set of unlicensed channels
*Ch_i,k_*(*t*)	Operation of *CB_i_* in channel *C_k_* at superframe *t*
*t_REQ_*	Length of REQ packet
*T_TEQ_*	Total length of REQ packets of *N* CRBANs
*t_SYN_*	Length of SYNC packet
Δ*T*(*CB_i_*)	*Start_time* or the start of superframe of *CB_i_*
*Ch_i,k_*(*t*) = 1	*CB_i_* occupies channel *C_k_* at superframe *t*
*SF*(*t,C_k_*)	Superframe length of channel *C_k_*
*T*(*CB_i_*)	Superframe length of *CB_i_*

**Table 2:** Simulation parameters.

Parameter	Value
Data slot time	10 ms
Number of PUs	9 (one PU per area)
Number of sensor per CRBAN	6
Number of CRBANs	36–72 (54 by default)
Priority value	1–3 (The highest priority is 3.)
Number of channels	4–7 (5 by default)
Transmitted power of CRBAN	10 dBm
Channel bandwidth	1 MHz
Transmit current	17.4 mA
Receive current	19.7 mA
Energy consumption per channel switching	2 mJ
Voltage	3.3 V
Receiver sensitivity	−80 dBm
